# PD45 - BCG vaccination and childhood asthma: preliminary results from the Québec Birth Cohort on Immunity and Health

**DOI:** 10.1186/2045-7022-4-S1-P45

**Published:** 2014-02-28

**Authors:** Marie-Claude Rousseau, Marie-Élise Parent, Dick Menzies, Andrea Benedetti, Florence Conus, Mariam El-Zein

**Affiliations:** 1 INRS-Institut Armand-Frappier, Laval, Canada; 2 McGill University Health Centre Research Institute, Montreal, Canada

## 

It remains unclear whether Bacillus Calmette-Guérin (BCG) vaccination is related to the development of childhood asthma. Meta-analyses on this topic have come to mixed conclusions. Between 1949 and 1974, a BCG vaccination program was conducted in Québec targeting neonates and school-age children. To investigate the effect of BCG vaccination on asthma, we assembled a retrospective cohort of subjects born in Québec in 1974 through the linkage of several administrative medical and demographic databases. BCG vaccination status, asthma-related events (medical services, hospitalizations, emergency department visits), and potential confounders were obtained for each subject until 1994. Logistic regression was used to estimate odds ratios (OR) and 95% confidence intervals (CI) adjusting for gender, birth weight for gestational age, number of older siblings, parents’ age, family income, and urban/rural residency. Among 90,060 individuals born in Québec in 1974, at or after 32 weeks of gestation, probabilistic linkage resulted in a cohort of 81,496 subjects (90.5%). Vaccination or asthma status could not be determined for 4,566 subjects. Among the remaining 76,930 subjects, 51% were males. Subjects vaccinated with BCG within their first year constituted 43% of the cohort, whereas those vaccinated later amounted to 3.5%. Those who had 2 or more asthma-related medical services or at least 1 hospitalization were considered asthmatics, representing 7.7% of the cohort. A slightly lower asthma risk was observed among those vaccinated as compared with non-vaccinated subjects; the adjusted OR was 0.94 (95% CI: 0.89, 0.99). The lack of information on some potential confounding factors is a limitation. Such information has been gathered by telephone interviews with a subset of subjects, and will allow further adjustment of the measure of association. So far, these preliminary results are in support of the immunological hypothesis that BCG vaccination in early life may influence immune maturation and prevent asthma.

